# Ventricular assist device implantation in a patient with systemic right ventricle and pectus excavatum

**DOI:** 10.1016/j.jccase.2022.03.021

**Published:** 2022-05-02

**Authors:** Mohammad Mostafa Ansari Ramandi, Stan A.J. van den Broek, Gianclaudio Mecozzi, Michiel E. Erasmus, Joost P. van Melle, Kevin Damman

**Affiliations:** a University of Groningen, Department of Cardiology, University Medical Center Groningen, Groningen, the Netherlands; b University of Groningen, Department of Cardiothoracic Surgery, University Medical Center Groningen, Groningen, the Netherlands

**Keywords:** Congenital heart disease, Heart failure, Transposition of the great arteries, HeartMate 3, VAD, Ventricular Assist Device, TGA, Transposition of the Great Arteries

## Abstract

Systemic right ventricular failure is a common finding in patients with transposition of the great arteries. Some of these patients require ventricular assist device implantation. We describe the feasibility of HeartMate 3 [Abbott, Illinois, United States] implantation in a patient with transposition of the great arteries, high human leukocyte antigen sensitization, and severe pectus excavatum using a two-stage approach.

**Learning objectives:**

1. To notice the challenges faced while implanting HeartMate 3 [Abbott, Illinois, United States] in patients with congenital heart disease and anatomical limitations.
2. To understand that despite the difficulties, HeartMate 3 implantation is possible, worthwhile, and sometimes the only choice in a patient with end-stage heart failure and congenital heart disease.

## Introduction

Patients with a systemic right ventricle, such as transposition of the great arteries (TGA) after Mustard correction, can experience advanced heart failure, eventually requiring heart transplantation [1]. Heart transplantation is not always an option due to the shortage of donor hearts, anatomical considerations, human leukocyte antigen (HLA) sensitization, and comorbidities. In particular, patients with (surgically corrected) congenital heart disease, who have had multiple operations in childhood, have a higher risk of unfavorable cardiac anatomy, as well as the risk of HLA sensitization [2]. Some of these patients may be suitable for ventricular assist device implantation, either as bridge to transplant or destination therapy (DT) [1].

## Case report

A 43-year-old Caucasian woman with history of TGA and Mustard procedure was referred to our heart transplant center for a second opinion because of progressive dyspnea and exercise intolerance as a result of end-stage heart failure. On physical examination, she had normal body mass index of 24.9 kg/m^2^, blood pressure of 105/61 mmHg, and normal temperature and respiratory rate. There were no significant murmurs, no leg edema, and normal breathing sounds. Her thorax was deformed by a severe pectus excavatum. Her past medical history is summarized in the timeline (Table 1).

**Table 1:** Patient timeline.

Childhood	Transposition of Great Arteries Pectus Excavatum Mustard Procedure
Adulthood	2 uncomplicated pregnancies
18 years prior	Transient ischemic attack
13 years prior	During 3rd pregnancy heart failure with systemic (right) ventricular failure and tricuspid regurgitation
9 years prior	Recurrent atrial tachycardias for which multiple cardioversions
9 years prior	Pulmonary embolism
8 years prior	Inferior baffle stenosis Re-sternotomy with widening of Mustard tunnel with Goretex patch.
3 years prior	Recurrent atrial tachycardias for which multiple cardioversions and amiodarone DDD-ICD implantation End-stage heart failure due to severe systemic (right) ventricular failure, severe tricuspid regurgitation. VAD deemed impossible. Referred to heart transplantation center for screening
2 years prior	Screening for heart transplantation, low PVR, high HLA sensitization (PRA 74%). Pectus Excavatum with severe acquired levocardia. After initial acceptance, declined for transplantation and referred to second heart transplant center
1–2 years prior	Second opinion: Heart transplantation deemed only possible in combination with correction of pectus excavatum. Due to reasonable aerobic capacity (17 mL/min/kg) watchful waiting
1 month prior	Re-evaluation due to NYHA IV, INTERMACS 4 heart failure. Aerobic capacity decreased to 13.4 mL/min/kg and she is wheelchair-bound. Multiple admissions for inotropes, high dose diuretics in referral hospital Re-screening for heart transplantation - Severe (initially) irreversible pre and post capillary pulmonary hypertension - Due to high HLA sensitization, pectus excavation, severe pulmonary hypertension declined for heart transplantation. - Accepted for VAD implantation (HeartMate 3) with minimal correction of pectus excavatum
Day of implantation	VAD implantation (HeartMate 3) in systemic right ventricle, lateral free wall.
4 days post	Completion of surgical correction pectus excavatum
27 days post	Discharge to rehabilitation center
64 days post	Discharged home
2.5 year post	NYHA II heart failure at home. Severe systemic AV valve regurgitation remains No unplanned readmissions in the first year post VAD implantation

DDD-ICD, dual chamber implantable cardioverter-defibrillator; HLA, human leukocyte antigen; ICD, implantable cardioverter defibrillator; INTERMACS, Interagency Registry for Mechanically Assisted Circulatory Support; NYHA, New York Heart Association; PRA, panel-reactive antibody; PVR, pulmonary vascular resistance; VAD, ventricular assist device.

## Investigations

Initial echocardiography showed a dilated systemic right ventricle with severe impairment. There was severe systemic atrioventricular (tricuspid) valve insufficiency, with a vena contracta of 10 mm. We could find no evidence of residual baffle obstruction (Video 1, Video 2).

On laboratory examination, she had a creatinine of 1.07 mg/dL (95 μmol/L), potassium of 3.5 mmol/L, and sodium of 137 mmol/L. The high-sensitivity C-reactive protein level was 8 mg/l and the N-terminal pro-B-type natriuretic peptide level was 5309 ng/L.

The electrocardiogram showed an AV-paced rhythm with wide QRS complexes with right bundle branch block and left anterior fascicular block with an extreme axis.

Initially, right heart catheterization showed severe combined pre- and post-capillary pulmonary hypertension, with low cardiac output based on a pulmonary capillary wedge pressure (PCWP) 26 mmHg, mean pulmonary artery pressure (mPAP) 54 mmHg, right atrial pressure (RAP) 18 mmHg and cardiac index (CI) 1.6 L/min/m^2^. Reversibility testing did reduce pulmonary vascular resistance (PVR) but increased the filling pressures, suggestive of severe systemic AV valve insufficiency in combination with ventricular failure.

**Fig. 1:**
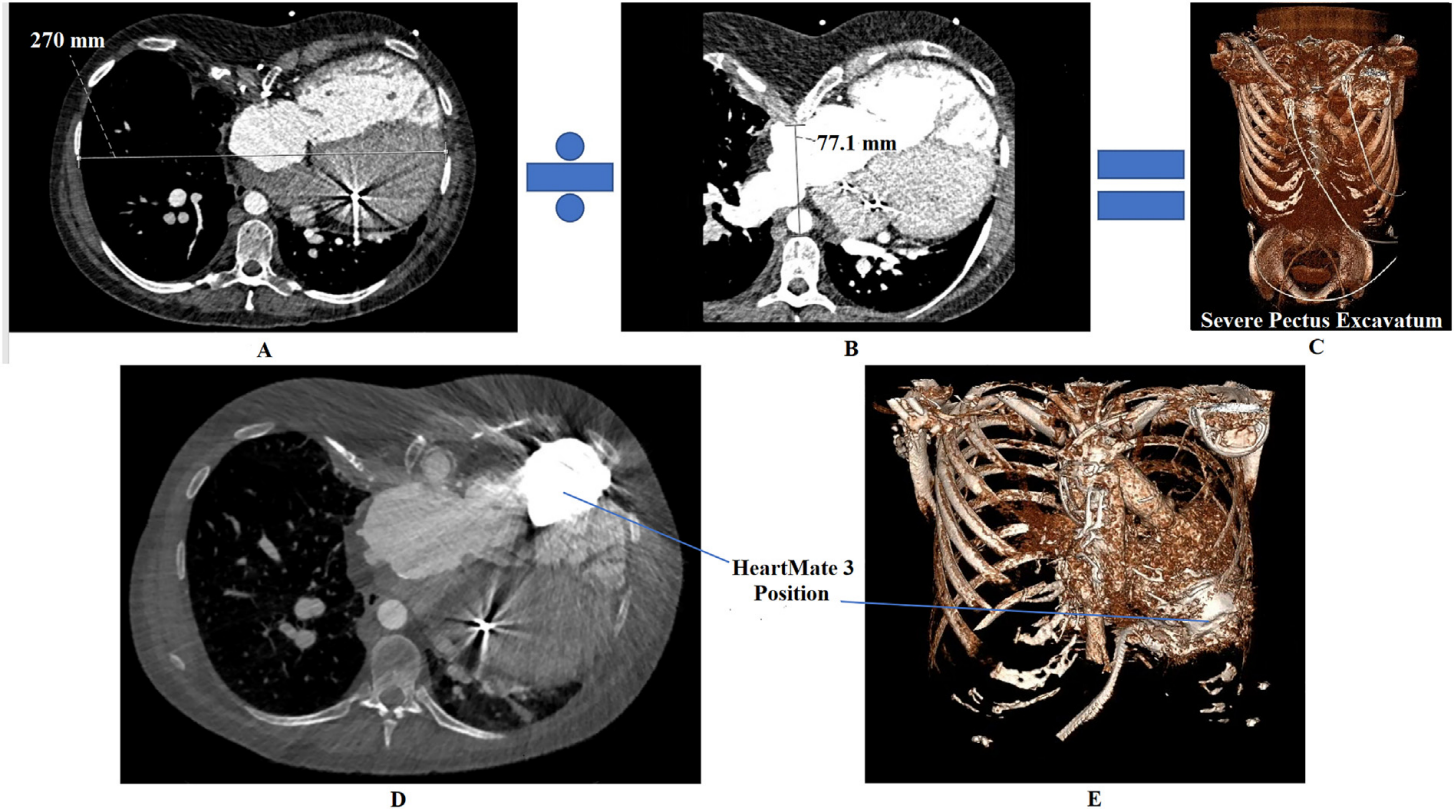
Pre- and post-procedural chest computed tomography (CT) scan. Pre-procedural chest CT scan: Calculation of Haller index in our patient by dividing the (A) transverse diameter of the chest by the (B) distance between the anterior angle of the spine and the posterior aspect of the deepest part of the sternum. Panel C shows the 3-dimensional image of the pectus excavatum in our patient. Postprocedural chest CT scan: Panel D shows position of the HeartMate 3 with respect to the sternum and the right ventricle and the 3-dimensional image for better demonstration is shown in panel E.

After intensification of high-dose loop diuretic therapy (bumetanide 10 mg/24 h) and treatment with dobutamine, a right heart catheterization was repeated which showed marked improvement (PCWP 13, mPAP 26 mmHg, RAP 8 mmHg, CI 3.1 L/min/m^2^), but after down-titrating of inotropes and intravenous diuretics, hemodynamics and renal function worsened. Computed tomography (CT) scan for determination of vascular access and extent of pectus excavatum showed absence of important collaterals, normal abdominal, and femoral access, and the known cardiac condition. Based on a Haller index of 3.5 (Fig. 1A–C), the pectus excavatum was considered severe [3], with also rotation of the sternum. Renewed HLA investigation showed a high degree of panel-reactive antibody (>75%, most conservative 84%, DR1, DR4, DQ7, DQ8, DQ10).

## Decision making

The patient was discussed in our advanced heart failure team and given the unfavorable anatomy, high degree of HLA sensitization, and severe pulmonary hypertension declined for heart transplantation at our center. She was accepted for ventricular assist device (VAD) placement in the systemic right ventricle as Destination Therapy (DT), together with minimal surgical correction of pectus excavatum to allow anastomosis of the outflow graft to the ascending aorta.

## Procedure

Before VAD placement, the patient was placed on cardiopulmonary bypass using a femoral approach for both arterial and venous access. To get access the distal sternum was mobilized by removing the cartilage and by transection below the manubrium followed by adhesiolysis of the heart, which was extremely displaced to the left thoracal cavity. Then the position for the inflow cannula in the systemic right ventricle was determined by visual inspection and transesophageal (TEE) assessment.

The most optimal position for the HeartMate 3 [Abbott, Illinois, United States] motor casing in the thorax was determined to be at the transition from the margo acutus to the right ventricular anterior wall. Some right ventricular trabeculae needed to be resected for optimal placement of the inflow cannula. The HeartMate 3 was also somewhat tilted by using asymmetric felt placement for a good position of the inflow cannula in regard to the interventricular septum and tricuspid valve. This was checked by TEE (Video 3, Video 4).

After 4 days (time taken to evaluate possible bleeding risk and need for reoperation) the pectus excavatum was partially corrected (cranial part) and the wound closed.

## Postoperative period

The early postoperative period was uneventful besides atrial tachycardias (for which cardioversion was carried out) and delirium. The CT scan of the patient is shown in panels D and E of Fig. 1. The patient was discharged from the intensive care to the coronary care unit on day 7, discharged to the ward on day 15, and to the rehabilitation facility on day 26.

The later inpatient postoperative period was remarkably stable, with normalized renal function. Initial VAD speed was set at 4800 rpm, and increased to 5000 rpm at day 1, and subsequently to 5200 rpm at day 8. This resulted in a pump flow of around 4.3 L/min. Transthoracic echocardiography showed normal pulmonary (left) ventricular function, a systemic (right) ventricular function that remained poor (Video 5) with also severe systemic AV valve insufficiency remaining. The inflow cannula was visible in the lateral free wall of the right ventricle, with unobstructed flow visible into the inflow cannula.

## Follow-up

Her exercise tolerance slowly increased in the first year after VAD placement. She feels comfortable at rest and mild to moderate exercise, can ride a bike and she has not been readmitted since discharge from the rehabilitation center. She has had no major adverse events since VAD implantation.

## Discussion

The management of heart failure due to failure of systemic right ventricles is complex. Heart transplantation and VAD implantation are challenging because of anatomical variations of the heart and great vessels, and HLA sensitization is frequent, limiting heart transplantation in these patients. We report the feasibility and safety of LVAD HeartMate 3 implantation for a patient with high HLA sensitization and pectus excavatum.

There have been limited reports on the implantation of VADs in systemic right ventricles, either in TGA or CCTGA, and both as a bridge to transplant or destination therapy [1,[4], [5], [6], [7]]. Regarding the type of VAD, less is known on the use of HeartMate 3 in such patients. We do know that the motor housing dimensions are slightly greater as compared with the Heartware HVAD [Medtronic, United States] (now discontinued), making positioning below the sternum somewhat more challenging.

The implantation procedure and the type of VAD are important factors for successful mechanical support in patients with systemic right ventricular failure. Other studies reporting the use of HeartMate 3 in TGA patients are summarized in Table 2 [1,[4], [5], [6]].

**Table 2:** Characteristics of TGA patients reported with HeartMate 3.

Author, publication year	Patient profile	Diagnosis	Previous surgical procedure (age)	VAD implant year	Techniques, procedural challenges, and possible consequences	Length on VAD	Outcome
Tadokoro N et al., 2020 [1]	34 years old male	ccTGA	Tricuspid valve replacement (2 years) *Re*-tricuspid valve replacement (16 years)	2020	HeartMate 3 was positioned anteriorly and towards the tricuspid valve	42 days	On VAD
Tadokoro N et al., 2020 [1]	40 years old male	TGA, VSD	Balloon atrial septostomy (1 month) Mustard, VSD closure (4 months) Pulmonary artery banding (22 years)	2020	The inserted VAD rotated the heart clockwise and deformed the subpulmonic LV structure. Although the LV hemodynamics were preserved, it may be a limiting factor in the long term of this VAD for TGA patients with a previous atrial switch.	70 days	On VAD
Gyoten T et al., 2020 [4]	35 years old male	ccTGA	Pulmonary artery banding (15 years)	2018	They used a beating heart technique for this patient, who then required additional temporary extracorporeal life support after the operation.	24 months	On VAD
White CW et al., 2020 [5]	46 years old male	TGA, VSD	Mustard, VSD closure (1st decade of life)	NA	The right ventricle was densely adherent to the sternum and it was thought that the sternum might push the inflow cannula deep in the ventricle. For this reason, they inserted the inflow cannula into the apex of the systemic right ventricle, using a left anterior thoracotomy and extensive resection of the right ventricular trabeculae.	NA	On VAD
Zhu A et al., 2020 [6]	36 years old female	TGA	Mustard (NA)	NA	They repaired an unrepaired partial anomalous pulmonary venous return and revised a Mustard baffle before implanting the HeartMate 3. This was done to prevent cyanosis due to intracardiac shunting and to reduce subpulmonic ventricular failure after the surgery.	NA	On VAD

ccTGA, congenitally corrected transposition of the great arteries; LV, left ventricle; NA, not available; TGA, transposition of the great arteries; VAD, ventricular assist device; VSD, ventricular septal defect.

The patient described in this case report had a severe pectus excavatum, which further compromised the available space for the motor housing of the HeartMate 3. We used pre-operative CT scanning to determine if there was enough space for VAD implantation and opted for a two-stage approach where at first a) the VAD was implanted in the systemic right ventricle and b) after 4 days (after stabilization and making sure no bleeding complications developed) a partial pectus correction (cranial part of the sternum) was carried out. To our knowledge, our case is the first report on the use of the HeartMate 3 in a patient with TGA and limited anatomical space due to pectus excavatum.

## Conclusions

This case showed that implantation of a VAD (HeartMate 3) is possible in patients with complex congenital anatomy, complicated by limited intrathoracic space due to pectus excavatum.

The following are the supplementary data related to this article.

Video 1 Initial transthoracic echocardiography showing a dilated systemic right ventricle with severe impairment. Video 1

Video 2 Initial transthoracic echocardiography showing severe systemic atrioventricular (tricuspid) valve insufficiency. Video 2

Video 3 Transesophageal echocardiography during procedure. Positioning of the inflow cannula in regard to the interventricular septum and tricuspid valve. Video 3

Video 4 Transesophageal echocardiography during procedure. Positioning of the inflow cannula in regard to the interventricular septum and tricuspid valve. Video 4

Video 5 Transthoracic echocardiography after the procedure showing normal pulmonary (left) ventricular function, a systemic (right) ventricular function that remained poor after ventricular assist device implantation. Video 5

## Declaration of competing interest

None.
